# Structurally Modified Bioactive Peptide Inhibits SARS-CoV-2 Lentiviral Particles Expression

**DOI:** 10.3390/pharmaceutics14102045

**Published:** 2022-09-26

**Authors:** Khushwant S. Bhullar, Manal A. Nael, Khaled M. Elokely, Steven J. Drews, Jianping Wu

**Affiliations:** 1 Department of Agricultural, Food & Nutritional Science, University of Alberta, Edmonton, AB T6G 2P5, Canada; 2 Department of Pharmacology, Faculty of Medicine and Dentistry, University of Alberta, Edmonton, AB T6G 2H7, Canada; 3 Institute for Computational Molecular Science and Department of Chemistry, Temple University, Philadelphia, PA 19122, USA; 4 Department of Pharmaceutical Chemistry, Faculty of Pharmacy, Tanta 31527, Egypt; 5 Canadian Blood Services, Department of Laboratory Medicine & Pathology, University of Alberta, Edmonton, AB T6G 2P5, Canada; 6 Cardiovascular Research Centre, University of Alberta, Edmonton, AB T6G 2R7, Canada

**Keywords:** COVID-19, peptides, SARS-CoV-2, ACE2, RBD, furin

## Abstract

Coronavirus disease 2019 (COVID-19), the current global pandemic is caused by severe acute respiratory syndrome coronavirus 2 (SARS-CoV-2). Various pharmaceuticals are being developed to counter the spread of the virus. The strategy of repurposing known drugs and bioactive molecules is a rational approach. A previously described molecule, Ile-Arg-Trp (IRW), is a bioactive tripeptide that exhibits an ability to boost angiotensin converting enzyme-2 (ACE2) expression in animals and cells. Given the importance of SARS-CoV-2 S receptor binding domain (RBD)-ACE2 interaction in SARS-CoV-2 pathophysiology, we synthesized various IRW analogs intending to mitigate the RBD-ACE-2 interaction. Herein, we describe two analogs of IRW, A9 (Acetyl-Ile-Arg-Trp-Amide) and A14 (Formyl-Ile-Arg-Trp-Amide) which lowered the SARS-CoV-2 S RBD-ACE2 (at 50 µM) in vitro. The free energy of binding suggested that A9 and A14 interacted with the SARS-CoV-2 S RBD more favorably than ACE2. The calculated MMGBSA ΔG of spike binding for A9 was −57.22 kcal/mol, while that of A14 was −52.44 kcal/mol. A14 also inhibited furin enzymatic activity at various tested concentrations (25, 50, and 100 µM). We confirmed the effect of the two potent analogs using SARS-CoV-2 spike protein overexpressing cells. Both peptides lowered the protein expression of SARS-CoV-2 spike protein at the tested concentration (50 µM). Similarly, both peptides, A9 and A14 (50 µM), also inhibited pseudotyped lentiviral particles with SARS-CoV-2 Spike in ACE2 overexpressing cells. Further, the molecular dynamics (MD) calculations showed the interaction of A9 and A14 with multiple residues in spike S1 RBD. In conclusion, novel peptide analogs of ACE2 boosting IRW were prepared and confirmed through in vitro, cellular, and computational evaluations to be potential seed candidates for SARS-CoV-2 host cell binding inhibition.

## 1. Introduction

The current coronavirus disease 2019 (COVID-19) outbreak since its beginning in Wuhan, China has become a global health emergency [1,2]. The causative novel virus, a *Betacoronavirus*, was named the severe acute respiratory syndrome coronavirus-2 (SARS-CoV-2 or 2019-nCoV) owing to its high homology (~80%) to another respiratory virus SARS-CoV [3]. As of September 2022, around 614,215,882 cases worldwide have been reported according to the John Hopkins University (Available on: https://coronavirus.jhu.edu/map.html, accessed on 23 September 2022). Clinically, the SARS-CoV-2 virus predominantly affects the pulmonary system but some evidence for active SARS-CoV-2 infection outside of the respiratory tract is slowly emerging [4,5,6]. COVID-19 in many cases results in severe respiratory illness such as an acute respiratory distress syndrome (ARDS) and multi-organ dysfunction, whereas cough, dyspnoea, accompanied by fever are the key initial symptoms of the disease [7]. Recent literature reports also indicate neurological symptoms such as headache, varied mental status, memory loss, depression, and anosmia, in many patients with COVID-19 [8,9]. However, in some cases, COVID-19 infection may even exhibit an asymptomatic state [10], while weaker pathogenesis has been observed in neonates, infants, and children as well [11,12]. Further, long-lasting COVID-19 or “long COVID” is characterized by parallel symptoms of fatigue, headache, dyspnea, and anosmia over a period of 4–12 weeks or more [13]. Apart from system level physiological changes, SARS-CoV-2 infection also impairs oxidative phosphorylation (OXPHOS), leading to augmented oxidative stress [14]. In our recent article, we summarized key clinical features of SARS-CoV-2 [15] and further seminal readings are available for detailed study as well [13,16,17].

The current clinical management of COVID-19 is largely supportive with no specialized targeted therapy, thus, overwhelming healthcare systems [18]. However, the recent discovery and characterization Paxlovid along with monoclonal antibodies and remdesivir may revolutionize COVID-19 treatment [19,20]. Further emergence of several variants of SARS-CoV-2, owing to intense pressure on the virus to genetically evolve in light of mRNA vaccination is another global challenge [21]. In a previous manuscript, we proposed developing peptide based pharmaceutics to inhibit specific SARS-CoV-2 pathophysiologic processes [15]. Some of the key pharmacological approaches included blocking the Angiotensin-Converting Enzyme 2 (ACE2), Transmembrane Serine Protease 2 (TMPRSS2), and furin processing by peptides substrates containing arginine, isoleucine, or leucine [15,22]. The scientific rationale behind the selection of these targets was based on the utilization of ACE2, TMPRSS2, and furin cleavage by SARS-CoV-2 spike (S) protein for cellular entry and processing [23,24]. Moreover, peptide substrates with arginine and hydrophobic isoleucine can bind to the large hydrophobic S1 pocket of TMPRSS2, possibly resulting in blockade of SARS-CoV-2 [25]. Likewise, arginine containing potent peptide based furin inhibitors have been developed [26,27]. Besides, peptides have several advantages over proteins or antibodies owing to their small size, easy synthesis, and cellular delivery through the membranes of cells [28]. 

**Figure 1:**
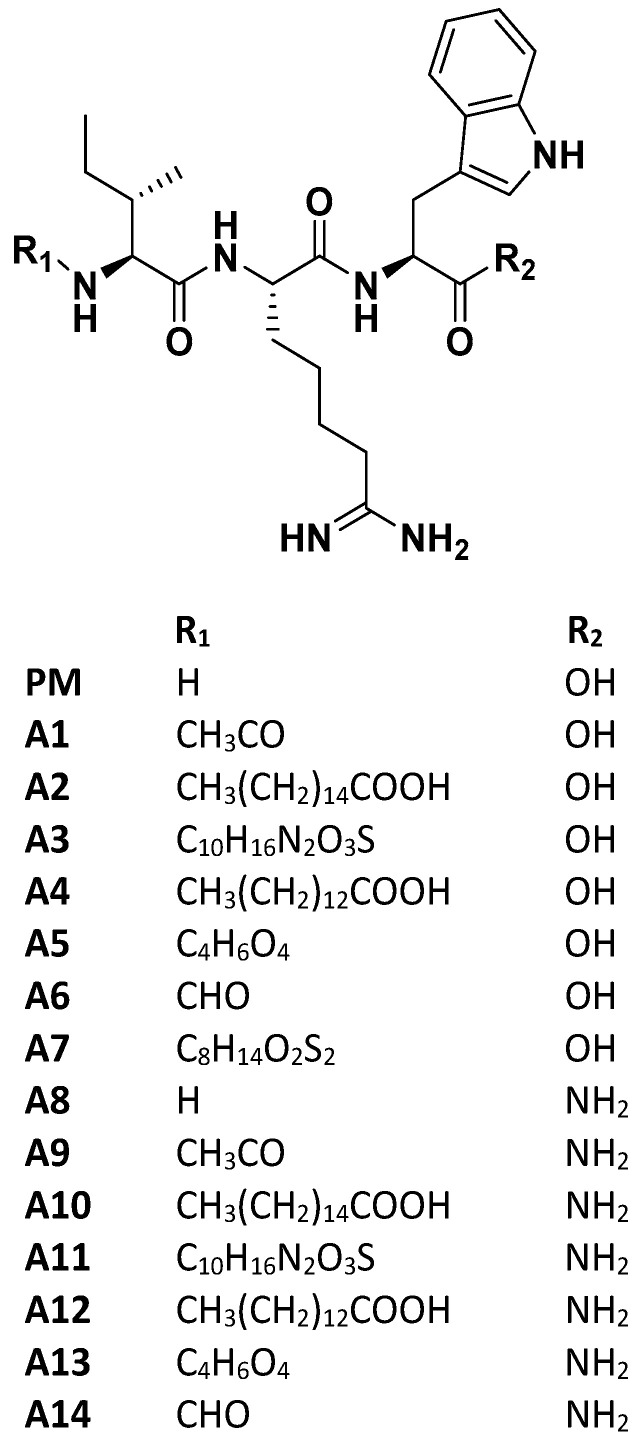
Design and chemical moieties of IRW analogs (A1–A14). PM: NH_2_-IRW-COOH A1: Acetylation-IRW-COOH; A2: Palmitic Acid-IRW-COOH; A3: Biotin-IRW-COOH; A4: Myristoyl-IRW-COOH; A5: Succinylation-IRW-COOH; A6: Formylation-IRW-COOH; A7: Lipoic acid-IRW-Amide; A8: NH_2_-IRW-Amide; A9: Acetylation-IRW-Amide; A10: Palmitic Acid-IRW-Amide; A11: Biotin-IRW-Amide; A12: Myristoyl-IRW- Amide; A13: Succinylation-IRW-Amide; A14: Formylation IRW-Amide. PM: Parent molecule; A1–A14: Analog1-Analog14.

Considering the critical need of therapeutics for the COVID-19 pandemic, drug or nutraceutical repurposing has gained impetus in medical research to find candidates for this new indication. Based on the idea that SARS-CoV-2 spike protein and ACE2 on the host cell surface is of great importance to initiate COVID-19 infection, we designed multiple analogs of ACE2 modulating hydrophobic peptide IRW (Ile-Arg-Trp) with unique terminal moieties. IRW has previously shown a spectrum of pharmacological properties including, anti-inflammatory, OXPHOS boosting, and metabolic boosting effects, both vitally beneficial in COVID-19 treatment [29,30]. Overall, our rationale for modification of IRW is threefold (1) N and C-terminal modifications are commonly used in peptide-based drug discovery to change the chemical properties of the parent molecule [31], (2) chemical modification(s) of a peptide can dramatically change its interaction with its receptor or other ligands (examples include oxytocin peptide [32]), this can apply directly to IRW as the modified IRW may act as a possible inhibitor of SARS-CoV2-ACE2 interaction, and (3) the antioxidant and protective effect of ACE2 against lung injury can be maintained by base skeleton of IRW in all analogs [29,33]. For the current study, 14 peptides were synthesized based on N- and C-terminal modifications of tripeptide IRW (Figure 1). Along with the rationale above, these analogs were expected to have higher lipophilicity, and/or improved proteolytic stability. All the candidate inhibitors were structurally stable and out of them, two peptides (A9 and A14) exhibited greater inhibitory activity against the spike protein expression and pseudotype lentiviral particles with SARS-CoV-2 Spike. Compared to the parent peptide (IRW), there is greater bioactivity of the two (selected) peptides, implying their potential as inhibitors of the SARS-CoV-2 infection. Apart from SARS-CoV-2, owing to cross-reactivity and structural similarity between human CoVs, these novel peptides may have strong potential on other coronavirus(es) infections [34]. Backed by further evidence-based research, these peptides certainly hold the potential to mitigate the ACE2−spike interaction-mediated SARS-CoV-2 infection. 

## 2. Materials and Methods

### 2.1. Materials

Tripeptide IRW and its analogs were synthesized by GenScript (Piscataway, NJ, USA). Peptide sequence and purity (99.8%) of the synthesized peptides were validated by high-performance liquid chromatography−tandem mass spectrometry (HPLC-MS/MS). Dulbecco’s modified Eagle’s medium (DMEM), fetal bovine serum (FBS), penicillin−streptomycin, 0.25% trypsin-ethylenediaminetetraacetic acid (EDTA), TRIzol, L-glutamine, MEM non-essential amino acids solution and phosphate-buffered saline (PBS) were purchased from Gibco/Invitrogen (Carlsbad, CA, USA). Antibodies reactive to ACE2 (ab108252), TMPRSS2 (ab92323), Furin (ab183495), recombinant Anti-SARS-CoV-2 Spike Glycoprotein S1 antibody (ab283942) and total OXPHOS Rodent WB Antibody Cocktail (ab110413) were obtained from Abcam, Inc. Thermo Fisher (Toronto, ON, Canada). The flag DYKDDDDK Tag Antibody (MA1-142-A488) was also obtained from Thermo Fisher (Toronto, ON). Allophycocyanin (APC) AffiniPure Goat Anti-Human IgG, Fcγ fragment specific antibody (109-135-098) was obtained from Jackson Labs (West Grove, PA, USA). Anti-rabbit IgG, HRP-linked antibody was obtained from New England Biolabs (Whitby, ON, Canada). Goat anti-rabbit IRDye 680RD secondary antibody and donkey anti-mouse 800CW secondary antibody were obtained from Licor Biosciences (Lincoln, NE, USA). Goat anti-rabbit IgG (H + L) secondary antibody AlexaFluor546 and rabbit anti-mouse IgG (H + L) secondary antibody AlexaFluor594 were purchased from Molecular Probes (Waltham, MA, USA). 

### 2.2. SARS-CoV-2 Spike-ACE2 Interaction Inhibitor Screening Assay

SARS-CoV-2 Spike-ACE2 Interaction Inhibitor Screening was performed using a commercial kit Cayman Chemical (502050, Ann Arbor, MI, USA) as per the manufacturer’s instructions. The potential inhibitors were identified using a recombinant rabbit Fc-tagged SARS-CoV-2 spike S1 RBD that binds to a plate precoated with a recombinant His-tagged ACE2 protein. A potential inhibitor interrupts this interaction and inhibition can be quantified by reading the absorbance at 450 nm. All the results were expressed as percentage inhibition with respect to the vehicle in which SARS-CoV-2 spike S1 RBD freely binds to ACE2, mimicking a vital feature of SARS-CoV-2 cellular infection. 

### 2.3. Molecular Docking

The Cryo-EM structure of ACE2 in complex with the RBD of SARS-CoV-2 [35] was downloaded from the protein data bank (PDB code: 7C8D; available at: https://www.rcsb.org/structure/7C8D, accessed on 10 August 2022). Protein preparation was processed in Maestro (by Schrödinger) using Protein Preparation Wizard as previously described [36] to adjust atom types, bond orders, add missing atoms, and fix incomplete loops with Prime [37,38]. Original hydrogens were deleted, and fresh ones were added. The hydrogen bonds were assigned for the amino acids at pH of 7.4. The protein structure was then subjected to restrained minimization using OPLS3e force-field [39]. The complex was then split into ACE2 and the RBD to be used for the docking experiment. The peptides were sketched in Maestro, and the 3D conformers was generated with acceptable bond length and angles using OPLS3e force-field. Prime and Protein Preparation Wizard were used for the preparation and minimization of the peptides. Peptide docking protocol was used to predict the binding mode of A9 and A14 in both ACE2 and the RBD [40]. The centroid of the docking grid was defined by the amino acids that constitute the interface between ACE2 and the RBD. MM-GBSA was used to rank the poses after docking.

### 2.4. Molecular Dynamics (MD) Simulations

Desmond software was used to perform the MD simulations with the default energy minimizations and heating algorithm. The top docking pose of A9 and A14 was then solvated in orthorhombic box using TIP3P water model using OPLS3e force-field parameters [41]. The length of covalent bonds was constrained by the SHAKE algorithm The length of covalent bonds was constrained by the SHAKE algorithm [42]. The system was equilibrated at 310 K using the Nose-Hoover thermostat and Martyna-Tobias-Klein piston barostat [43]. The particle-mesh Ewald method was used for the long-range electrostatic interactions, and the cut-off distance of the non-bond interaction was considered as 9Å. MD was carried out for 100 ns using NPT ensemble with 310 K using OPLS3e force field. MM-GBSA calculations were performed to compute the average free energy of 20 representative clusters for A9 and A14 complexes (obtained by running the Desmond trajectory clustering algorithm).

### 2.5. Cell Culture

Cell culture was performed according to our recent report [44]. Briefly, HEK293T cells (ATCC^®^ CRL-3216™; transformed embryonic human kidney) were purchased from ATCC (American Type Culture Collection) and cultured in DMEM medium supplemented with 10% FBS, L-Glutamine, MEM non-essential amino acids solution, and 100 units/mL penicillin–streptomycin at 37 °C and 5% CO_2_ in an incubator. All cells were initially cultured in Corning^®^ T-75 flasks (catalog #430641) and growth medium was replaced every 48 h. Peptides were suspended in nuclease-free water to obtain stock solutions of 50 mM (10^−3^ mol/L) and their toxic impact was checked by cell viability assay using cell counting slides for TC10™ Cell Counter (Biorad, Mississauga, ON, Canada).

### 2.6. Immunoblotting

Immunoblotting was performed according to our recent report [29]. Briefly, HEK293T cells were grown in 6-well tissue culture plates until they reached ~80% confluency. They were then treated with 50 μM IRW or its analogs for 24 h. Following 24 h incubation, the culture medium was carefully removed, and the cells were lysed in RIPA buffer. These cell lysates were run on sodium dodecyl sulphate–polyacrylamide gel electrophoresis (SDS-PAGE), transferred to nitrocellulose membranes, blocked in tris buffered saline with 5% low-fat milk (TPBS) solution, and immunoblotted with primary antibodies in 1:500 concentration. Next, after incubating overnight with primary antibodies, blots were washed with TPBS and incubated with the appropriate secondary antibodies. Finally, after washing excess secondary off with TPBS, the antibody reactive protein bands were detected using a Licor Odyssey BioImager (Licor Biosciences, Lincoln, NB, USA) and quantified by densitometry using Image Studio Lite 5.2 software (Licor Biosciences, Lincoln, NB, USA). 

### 2.7. ACE2 Measurement Assay

An angiotensin II Converting Enzyme (ACE2) Assay Kit (Fluorometric) was employed to measure the amount of ACE2 in cells (ab273297, Abcam, Toronto, ON, Canada). The experiment was performed according to the manufacturer’s instructions and results were expressed as nmol of ACE2 per µL of cell extract.

### 2.8. SARS-CoV-2 S Overexpression

The pCMV14-3X-Flag-SARS-CoV-2 S was a gift from Zhaohui Qian and coworkers (Addgene plasmid # 145780; http://n2t.net/addgene:145780, accessed on 10 August 2022; RRID:Addgene_145780; Addgene, Watertown, MA, USA) and transfection was performed according to the methodology described in their report [45]. 

### 2.9. Enzyme Inhibition Assays

The in vitro furin inhibition assay was performed using a SensoLyte^®^ Rh110 furin activity fluorometric assay kit (AS-72256, Anaspec, Fremont, CA, USA). The potential peptide based furin inhibitors were assessed based on their ability to interrupt furin activity using a fluorogenic substrate. Upon cleavage by furin, the kit substrate generates the Rh110 (rhodamine 110) fluorophore with bright green fluorescence that can be detected at excitation/emission = 490 nm/520 nm. The results were expressed as percentage inhibition with respect to the vehicle control (nuclease free water) in which furin freely cleaves its substrate, a physiological feature of SARS-CoV-2 cellular replication. The SARS-CoV-2 3CL protease inhibition assay was performed according to a recent report by Akaberi and coworkers highlighting the mitigation of the replication of SARS-CoV-2 by nitric oxide in vitro [46]. Briefly, SARS-CoV-2 3CL Protease (aa1-306) (RP-87698, Thermo Fisher, Toronto, ON, Canada), at a final concentration of 100 nM, was incubated with selected peptides at varying concentrations in assay buffer (20 mM Tris-HCl pH 7.5, 0.01% Triton X-100) for 15 min at room temperature. The FRET substrate DABCYL-Lys-Thr-Ser-Ala-Val-Leu-Gln-Ser-Gly-Phe-Arg-Lys-Met-Glu-EDANS (M-2575, Bachem Holding AG, Bubendorf, Switzerland) was then added at a final concentration of 25 µM to start the enzymatic reaction. The selected peptides were solubilized in nuclease free water and the latter was used as the vehicle control. The fluorescence emission was monitored every 60 s for 35 min at 37 °C at the excitation wavelength of 355 nm and the emission wavelength of 538 nm. The results were expressed as % protease activity inhibition, with respect vehicle. 

### 2.10. SARS-CoV-2 Spike-Pseudotyped Lentiviral Particles Assay

The use of spike protein lentivirus assay has been recently reported as a novel method to conveniently measure Spike mediated cell entry via fluorescent or luciferase reporters [47]. We used the methodology described in this paper and BEI SARS-Related Coronavirus 2, Wuhan-Hu-1 Spike D614G-Pseudotyped Lentiviral Kit (NR-53817; BEI Resources, Manassas, VA, USA) to conduct the lentivirus study. The initial transfection was conducted using Viral Entry Protein (S D614G Glycoprotein, NR-53765), Lentiviral Backbone (Luc2; ZsGreen; NR-52516; fluorescent), and helper Plasmids (Gag; pol, NR-52517; Tat1b, NR-52518; Rev1b, NR-52519). These plasmids contain the beta-lactamase gene, *bla*, and were transformed through ampicillin resistance in One Shot™ TOP10 Chemically Competent *E. coli* (Invitrogen, Carlsbad, CA, USA) according to the manufacturer’s instructions. The spike lentivirus was produced in 293T cells and were harvested after 24 h of transfection. These lentiviruses expressing spike successfully infected cells that overexpressed the ACE2 receptor [293T-ACE2.TMPRSS2 (mCherry) cells] and induced fluorescent expression of ZsGreen as infection indicator. The change in expression of lentivirus production in cells following co-treatment with the selected IRW analogs (A9 and A14, 50 μM for 24 h) was measured using fluorescence microscopy (Leica DMRXA Microscope, Concord, ON, Canada) and flow cytometry analysis (The BD FACSAria III cell sorter, Mississauga, ON, Canada). 

### 2.11. Statistical Analysis

All data are presented as mean ± standard deviation (SD) of minimum three independent experiments. All statistical analyses were performed using GraphPad Prism software version 5.02 (GraphPad Software, San Diego, CA, USA). Statistical difference was determined by using one-way ANOVA followed Bonferroni’s post hoc test for the vehicle. *p* < 0.05 was considered significant. 

## 3. Results

### 3.1. SARS-CoV-2 Spike S1 RBD-ACE2 Interaction Inhibition

**Figure 2:**
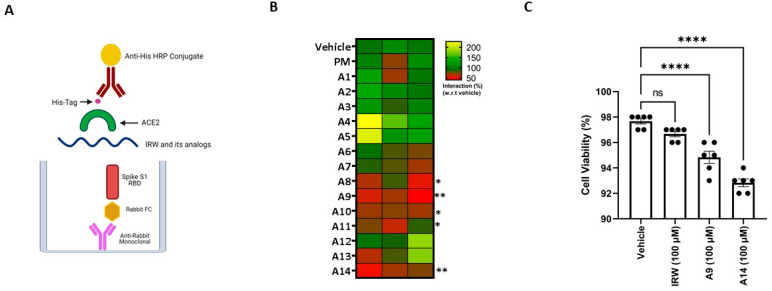
Impact of IRW and its analogs on SARS-CoV-2 S1 RBD-ACE2 interaction. (**A**) An in vitro assay was conducted to assess the possible (**B**) inhibitory impact of IRW and its analog peptides (50 µM) on SARS-CoV-2 S1 RBD-ACE2 interaction and (**C**) toxicity analysis of the selected peptides. The results are expressed as percentage inhibition with respect vehicle control (nuclease free water). Data expressed as mean ± SEM of n = 3–6. Experiments were reproduced three times with independent tips and samples. *p* values were determined by Analysis by one-way ANOVA followed by Bonferroni’s post hoc test for the vehicle. * *p* < 0.05, ** *p* < 0.01, **** *p* < 0.001 versus vehicle and ns: nonsignificant.

**Figure 3:**
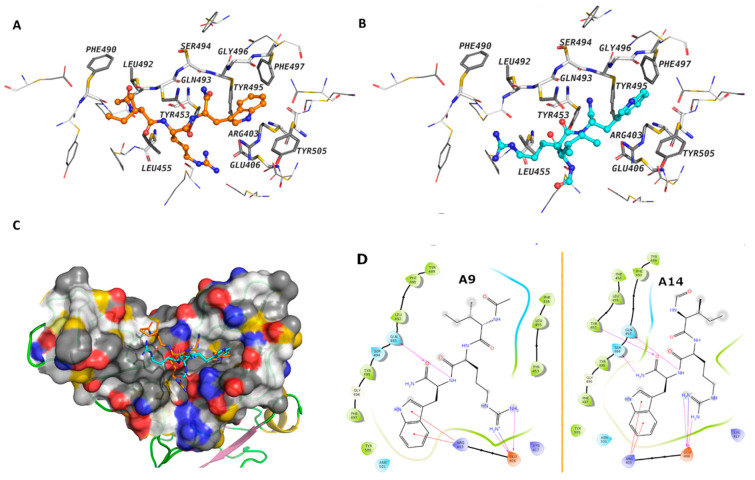
The binding modes of peptides A9 and A14. (**A**,**B**) A9 and A14 are shown as balls and sticks, the amino acids that are within 4Å of the peptides are labeled and shown as sticks (**C**) the surface cavity of the RBD is shown as molecular surface and (**D**) The 2D interaction diagram of A9 and A14. The surface is colored with the YRB scheme, yellow for the carbon atoms with high potential to form hydrophobic interactions, blue for the sidechain nitrogen atoms of arginine and lysine, and red for the sidechain oxygen atoms of glutamate and aspartate.

The SARS-CoV-2 spike S1 RBD-ACE2 interaction assay showed that structural modification of the parent molecule, IRW (Figure 1), leads to inhibition of Spike S1 RBD and ACE interaction in vitro (Figure 2A). Compared to the IRW, its five analogs, A8, A9, A10, A11, and A14 (50 µM) exhibited significant inhibition of SARS-CoV-2 spike S1 RBD-ACE2 interaction (Figure 2B). The 50 µM concentration was selected as per our recent reports showing impact of IRW on ACE2 and other pharmacological targets [29,48,49]. Among the five pharmacologically active analogs, the SARS-CoV-2 spike S1 RBD-ACE2 interaction was mitigated by A9 (55%, Acetyl-Ile-Arg-Trp-Amide) and A14 (63%, Formyl-Ile-Arg-Trp-Amide) were the strongest in vitro (*p* < 0.01). However, constituent dipeptide combinations of ARW, IAW, and IRA failed to inhibit the SARS-CoV-2 spike S1 RBD-ACE2 interaction in vitro (Figure S1). Based on the inhibition activity results, two analogs of IRW, A9 and A14 were selected for further experimentation. Additionally, their toxicity analysis showed that all cells treated with both peptides (100 µM) exhibited 92–97% cell viability (Figure 3C), thus exhibiting a safe profile for further analysis.

### 3.2. Molecular Interaction of Active Peptides with SARS-CoV-2 Spike RBD

Molecular docking was conducted to gain insights into the mechanistic interaction of A9 and A14 with the SARS-CoV-2 Spike S1 RBD and ACE2. The MM-GBSA binding energy showed better affinities of A9 and A14 over other analogs (Table 1), which is consistent with our experimental findings. The free energy of binding suggested that A9 and A14 interact with the RBD more favorably than with ACE2 (Table 1). In case of the spike protein, the calculated MMGBSA Δ*G* of binding for A9 was predicted to be –57.22 kcal/mol, while that of A14 was –52.44 kcal/mol. The average MM-GBSA binding energy of the clusters after MD simulations showed very similar free binding energy for A9 of –35.55 kcal/mol and for A14 of –32.88 kcal/mol. The Trp and Arg amino acids of A9 and A14 interacted with the surface amino acid residues of the spike protein through several electrostatic interactions, while the Ile displayed hydrophobic contacts (Figure 3A,B). A9 showed cation-π interactions between the indole ring of the Trp residue and Arg403, and hydrogen bonds between the backbone and Gln493 (Figure 3A). The Arg displayed hydrogen bonds and ionic interactions with Glu406. The Ile amino acid of the peptides interacted with hydrophobic contacts with Leu492, Phe490, Tyr489, Phe456, Leu455, and Tyr453 of the RBD domain. These interactions were observed in both IRW analogs (Figure 3D). In case of ACE2 (Figure S5), the calculated MMGBSA Δ*G* of binding for A9 and A14 at the surface cavity that constitutes interface with spike, was predicted at the level of –40.88 kcal/mol and –35.51 kcal/mol; respectively. The terminal amino acids of A9 and A14 did not show strong interactions with the surrounding amino acids. These results indicated that IRW analogs, A9 and A14 exhibited strong RBD interaction, suggesting inhibitory activity.

### 3.3. Impact of Selected Peptides on Cellular Levels of ACE2 

**Figure 4:**
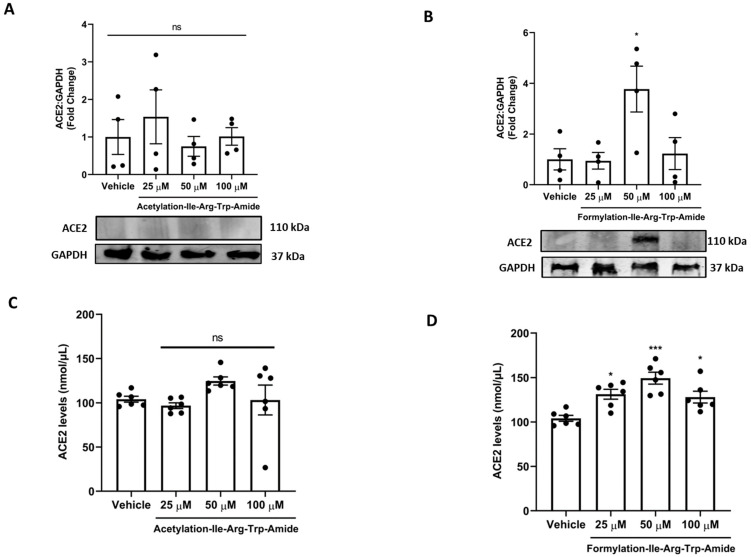
Impact of peptides A9 and A14 on ACE2 levels in cells. (**A**,**B**) Immunoblots showing the changes in ACE2 levels of HEK293T cells after treatment with A9 and A14 at the different tested concentrations and (**C**,**D**) impact of A9 and A14 on cellular levels of ACE2 using an activity assay Kit (ab273297, Abcam, Toronto, ON). HEK293T cells were grown in DMEM complete media containing 10% FBS with antibiotics. The cells were treated with vehicle (nuclease free water) or A9 or A14 at different concentrations (25, 50, and 100 µM) for 24 h. Thereafter, protein was extracted using RIPA buffer and was stored at −20 °C till further analysis. For immunoblotting, the results are expressed as change in ACE2 fold change with respect vehicle control (nuclease free water). For ACE2 activity assay, the results were expressed as nmol of ACE2 per µL of cell extract. Data expressed as mean ± SEM of n = 4–6. *p* values were determined by Analysis by one-way ANOVA followed by Bonferroni’s post hoc test for vehicle. * *p* < 0.05 and *** *p* < 0.001 indicate versus vehicle. The error bar indicates S.D. of the mean value. µM: micromolar, ns: nonsignificant.

Next, we assessed if A9 and A14 had impact on ACE2 in cells in vitro (Figure 4). Our results showed that A9 had no impact on cellular ACE2 levels, as shown by both immunoblotting and ELISA results (Figure 4A,C). However, A14 (50 µM) significantly increased ACE2 levels in cells as shown by immunoblotting (*p* < 0.05) and ELISA (*p* < 0.001) in vitro (Figure 4B,D). This was an interesting finding as ACE2 binding MMGBSA Δ*G* for A9 and A14 was predicted at the level of –40.88 kcal/mol and –35.51 kcal/mol. A more negative value indicates that more spontaneous binding occurs between a ligand to a protein receptor and vice versa. Hence, acetylated IRW (A9) looses its ability to increase ACE2, while formylated IRW (A14) preserves its ability to increase ACE2. However, at a higher concentration of 100 µM, the A14 peptide treatment resulted in a decline in ACE2 levels, possibly due to aggregate formation at a higher concentration, indicating the role of peptide concentration in the pharmacological stability of peptides (Figure 4B,D). 

### 3.4. Impact of Selected Peptides on Cellular Levels of TMPRSS2 and Furin 

**Figure 5:**
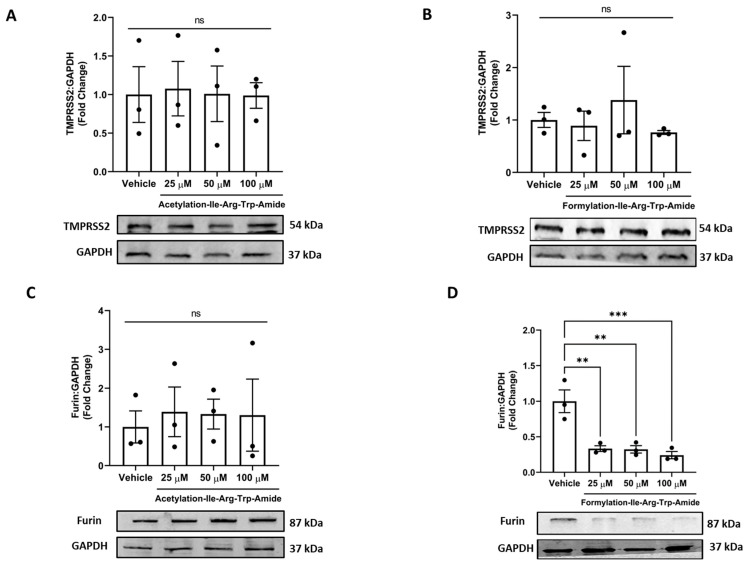
Impact of peptides A9 and A14 on TMPRSS2 and Furin levels in cells. Immunoblots showing the changes in (**A**,**B**) TMPRSS2 and (**C**,**D**) furin levels of HEK293T cells after treatment with A9 and A14 at the different tested concentrations. HEK293T cells were grown in DMEM complete media containing 10% FBS with antibiotics. The cells were treated with vehicle (nuclease free water) or A9 or A14 at different concentrations (25, 50, and 100 µM) for 24 h. Thereafter, protein was extracted using RIPA buffer and was stored at −20 °C till further analysis. The results are expressed as change in TMPRSS2 and furin fold change with respect vehicle control (nuclease free water). Data expressed as mean ± SEM of n = 3. *p* values were determined by Analysis by one-way ANOVA followed by Bonferroni’s post hoc test for vehicle. ** *p* < 0.01 and *** *p* < 0.001 indicate versus vehicle. The error bar indicates S.D. of the mean value. µM: micromolar, ns: nonsignificant.

Our results showed that both analogs had no impact on TMPRSS2 levels in cells at the tested concentrations, an essential host factor for SARS-CoV-2 cell entry and pathogenicity (Figure 5A,B). Among the two analogs, analog A14 (25, 50, and 100 µM) had a significantly decreased furin levels, indicating furin inhibition in cells (Figure 5D). In contrast, the analog A9 had no impact on furin inhibition in cells (Figure 5C). This supports the ability of A14 to inhibit SARS-CoV-2 infection as the cleavage of the spike at the S1/S2 site after receptor interaction is impeded [50]. However, both analogs (50 µM) significantly inhibited furin using a peptide substrate assay in vitro (*p* < 0.001) (Figure S2). 

### 3.5. Selected Peptides Inhibit SARS-CoV-2 S Protein Expression

**Figure 6:**
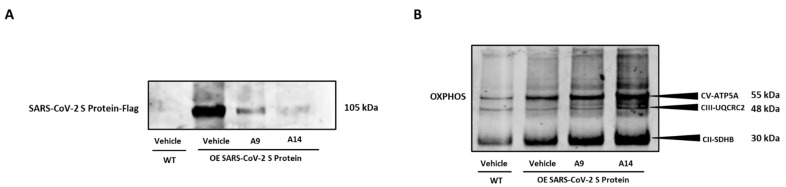
Impact of peptides A9 and A14 on over expressing SARS-CoV-2 S (spike) protein in HEK293T cells. Immunoblots showing the changes in (**A**) SARS-CoV-2 S (spike) and (**B**) OXPHOS levels of HEK293T cells after treatment with A9 and A14 at the tested concentration. HEK293T cells were grown in DMEM complete media containing 10% FBS with antibiotics. The SARS-CoV-2 S (spike) protein was overexpressed in cells. These cells were treated with vehicle (nuclease free water) or A9 or A14 at 50 µM for 24 h. Thereafter, protein was extracted using RIPA buffer and was stored at −20 °C till further analysis. These experiments were repeated thrice independently. µM: micromolar.

Following assessment of various molecular factors as shown above, we inquired if the selected IRW analogs, A9 and A14 could inhibit overexpression spike S1 protein in HEK293T cells. Our results showed that both analogs, A9 and A14 (50 µM), inhibited the protein expression of spike S1 protein in HEK293T cells (Figure 6A). These results supported our in vitro antibody assay and docking studies as shown above (Figure 2B and Figure 3A,B). Additionally, we observed a significant increase in the markers of OXPHOS in spike S1 protein overexpressing HEK293T cells by treatment of IRW analogs, A9 and A14 (Figure 6B). These results showed that analogs A9 and A14 (50 µM) managed to inhibit overexpression spike S1 protein in HEK293T cells and boost their redox status (Figure 6A,B). 

### 3.6. SARS-CoV-2 Spike-Pseudotyped Lentiviral Particles Assay

**Figure 7:**
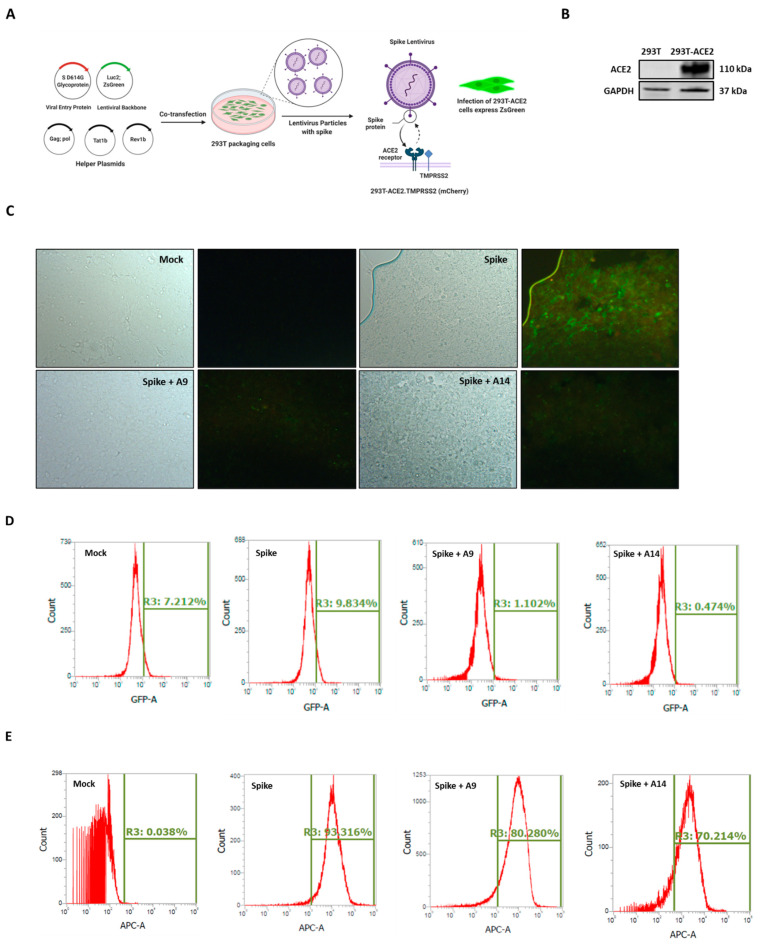
Impact of peptides A9 and A14 on SARS-CoV-2 Spike-pseudotyped Lentiviral Particles. Lentiviral pseudotyping schematic showing the use of (**A**) 293T cells as packaging cells for producing lentivirus particles using Viral Entry Protein (S D614G Glycoprotein), Lentiviral Backbone (Luc2; ZsGreen), and helper plasmids expressing the other HIV proteins needed for virion formation (Tat, Gag-Pol, and Rev). The transfected 293T cells produce lentiviral particles with surface Spike and can infect (**B**) 293T-ACE2.TMPRSS2 (mCherry) cells (**C**) Bright-field and fluorescence microscopy images; and (**D**) flowcytometry analysis showing changes in ZsGreen expression in 293T-ACE2.TMPRSS2 cells at 24 h after incubation with Spike-pseudotyped lentiviral particles with the ZsGreen backbone in presence of A9 and A14 at 50 µM and (**E**) the spike lentivirus count in the 293T-ACE2.TMPRSS2 cells transfected with the Spike lentiviral particles with the ZsGreen backbone in presence of A9 and A14 at 50 µM. Flow cytometry was used to measure Spike by staining with Recombinant Anti-SARS-CoV-2 Spike Glycoprotein S1 antibody followed by staining (1:500 dilution) with an APC AffiniPure Goat Anti-Human IgG, Fcγ fragment specific antibody (Jackson Labs, 109-135-098) (1:100 dilution).

The spike lentivirus entry assay as conducted as shown in Figure 7A using 293T-ACE2.TMPRSS2 (mCherry) cells, with abundant ACE2 and TMPRSS2, the receptor for viral entry and the serine protease for spike protein priming, respectively (Figure 7B) [22]. Our microscopy results showed that induction of SARS-CoV-2 spike-pseudotyped lentiviral particles significantly increased the overexpression of green fluorescent protein (GFP) in cells (Figure 7C). The increased GFP confirmed the infection of 293T-ACE2 cells with lentivirus particles. In line with our protein expression findings (Figure 6A), the co-treatment of cells with A9 and A14 (50 µM), strongly allayed the expression of GFP in 293T-ACE2 cells (Figure 7C and Figure S4). These findings were then confirmed using flow cytometry analysis (FITC screening). Similar to microscopy results, both IRW analogs, A9 and A14 decreased the levels of GFP expression compared to the spike infection (1.102% and 0.474% vs. 9.834%) (Figure 7D). These findings showed that both the compounds could inhibit lentivirus growth in vitro. However, the direct measurement of lentivirus particles is key for validation of both microscopy and flowcytometry findings on GFP expression in 293T-ACE2 cells (Figure 7C,D). Our flowcytometry analysis, using SARS-CoV-2 Spike glycoprotein S1 subunit for detection of lentivirus showed a strong increase in spike lentivirus in spike group compared to the control group (Figure 7E). Similar to results from florescence microscopy and flowcytometry analysis for GFP detection), the co-treatment of cells with A9 and A14 (50 µM), inhibited the production of spike lentivirus in 293T-ACE2 cells (Figure 7E). These results confirmed the ability of two novel peptides A9 (Acetyl-Ile-Arg-Trp-Amide) and A14 (Formyl-Ile-Arg-Trp-Amide) to inhibit SARS-CoV-2 Spike-pseudotyped lentiviral particles in cells. 

### 3.7. MD Analysis

**Figure 8:**
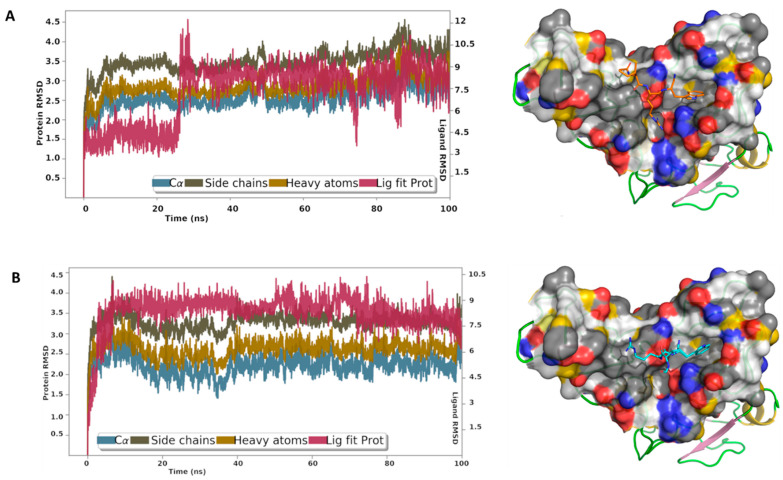
The root mean square deviation curves of A9 and A14 complexes with the RBD. The initial system is displayed on the left showing (**A**) A9 and (**B**) A14 in the binding site of the RBD. YRB scheme was used to highlight the hydrophobic regions. The plot shows the RMSD of the Ca, side chains, and heavy atoms of the RBD. ‘Lig fit Prot’ displays the RMSD of a ligand after aligning the protein-ligand complex on the protein backbone of the initial configuration and measuring the RMSD of the ligand-heavy atoms.

Finally, to gain more insights into the binding interactions of these peptides with the spike S1 RBD, MD simulations were carried out for the best docking poses of peptides A9 and A14 (Figure 8). The root mean square deviation (RMSD) reflected the stability of the peptides in the binding site, particularly after a short period of equilibration. The RMSD is used with respect to the first frame to measure the average displacement change of the atoms of the peptides and the protein. The protein interactions with A9 and A14 were monitored throughout the simulation time. Our results showed that A9 interacts via direct hydrogen bonds to Ser494, Tyr453, and Glu406, and through water-bridged hydrogen bonds to Asn501, Arg403, and Glu406 (Figure 8A). Internal hydrogen bond between Trp and Ile was monitored for about 18% of the 100 ns MD simulations time. The indole ring was involved in π-π contacts with Tyr505 and cation-π contacts with Arg403. A14 displayed direct hydrogen bonds with Ser494, Tyr453, Ala520, His519, and Gln493, and through water bridges with Asn501, and Arg403 (Figure 8B). The Trp residue in A9 and A14 was most stable in its position, while the Ile and Arg fluctuated to some extent. The acetyl cap suffered less from the desolvation penalty due to the hydrophobic nature of the methyl group, while the formyl cap is less hydrophobic and expected loss in the binding energy because of the solvation effect. The MD analysis showed us in depth, the mechanistic phenomenon involved in interaction of A9 and A14 with spike S1 RBD. Finally, the computed RMSD of A9 and A14 relative to the initial configuration (Figure S6), revealed high stability of the conformational state of the peptide throughout the entire MD simulations.

## 4. Discussion

The global spread of SARS-CoV-2 and mounting cases of COVID-19 necessitates the study of potential therapeutic agents (https://www.fda.gov/drugs/coronavirus-covid-19-drugs/coronavirus-treatment-acceleration-program-ctap, accessed on 10 August 2022). The genetic sequencing of SARS-CoV-2 and the study of the pathophysiology of COVID-19 has triggered intense global research efforts to develop pharmaceuticals to counter the disease. A striking feature of the drug development for COVID-19 is the range of research platforms being explored, including the development of repurposed drugs and bioactives against COVID-19 [51]. Rationale bioactive repurposing, owing to its low toxicity, can provide targeted drug candidates in a relatively short period. Given that the inhibitors of SARS-CoV-2 can aim at multiple targets such as RBD, ACE2 TMPRSS2, furin, and others, the bioactive repurposing effort has a consistent wider range and probability of success [15]. Our findings show that chemical modulation of IRW, an ACE2 activating tripeptide, can have a direct impact on the SARS-CoV-2 RBD. Structurally modified IRW, A9 (Acetylation-Ile-Arg-Trp-Amide) and A14 (Formylation-Ile-Arg-Trp-Amide) inhibited the interaction in situ and SARS-CoV-2 S Protein expression in HEK293T cells (Figure 2B and Figure 6A). The lowering of SARS-CoV-2 S Protein expression is a vital pharmacological aspect of the modified peptides, A9 and A14. This feature of both peptides indicates their ability to possibly counter viral infection by inhibiting SARS-CoV-2’s virion envelope involved in the receptor recognition [52]. These structural modification(s) led to the efficient free energy of binding leading to favorable interaction with the SARS-CoV-2 RBD.

To obtain IRW analogs with high biostability, chemical modifications at its N- and C-terminus, including acetylation, formylation, fatty acid conjugation, glycosylation, and others, were carried out (Figure 1). Among these modifications, N-terminal acetylation, and formylation with the addition of the C-terminus amide group boosted the pharmacological impact. The acetylation of the bioactive compounds may favor the in vivo biological activity by increasing their hydrophobicity and cellular uptake [53]. It also makes the peptide closely mimic the charge state in the native protein [54]. A recent report has shown that N-terminal acetylation significantly enhanced the antimicrobial activity of peptide L163 against multiple pathogens [55]. Likewise, N-terminal acetylation of peptide cathelicidin LL-37 improved its antimicrobial activity as well [56]. N-formylated peptides play an important role in host defense against microbial agents via modulation of the immune system [57]. Therefore, the N-terminal formylation of IRW boosted its ability to interact with SARS-CoV-2 RBD. Another consideration is the addition of C-terminal amidation, a common feature of wild type membrane disrupting antimicrobial peptides [58]. C-terminus amidation stabilizes α-helical confirmation by additional hydrogen [59,60]. This modification can yield a higher cationic charge leading to enhanced peptide binding with membranes of microbes [61]. Further, this modification makes the resulting peptide more stable towards enzymatic degradation resulting from exopeptidases [62,63]. Further, it is critical to mention that A1 and A6 peptides without C-terminal amide moiety failed to elicit any bioactivity even in presence of N-terminus N-terminal acetylation, and formylation (Figure 1). Hence, the collective alteration of terminal groups of IRW leads to potent changes in its pharmacological activity. Previous studies have also shown that antimicrobial activity of peptides (e.g., dermaseptin s3) is enhanced by multiple folds with the addition of an amide group instead of a free carboxylic acid at the C-terminus [64]. 

Our findings are in line with many recent studies to combat SARS-CoV-2 infection. Kobophenol A, a tetramer of resveratrol, blocked the interaction between the ACE2 receptor and S1-RBD in vitro [65]. Quinolines and terpenes based inhibitors of Spike-RBD-ACE2 were identified using methods similar to ours [66,67]. In another report, 3800 FDA approved drugs were screened for their ability to inhibit the RBD-ACE2 interface as a target [68]. Rationally designed, small peptide inhibitors with the ability to block the interaction of SARS-CoV-2 spike protein with ACE2 have been developed as well [69]. A study has shown that daily intranasal administration lipopeptide fusion inhibitors completely stopped the SARS-CoV-2 direct-contact transmission in animals [70]. EK1C4, a lipopeptide derived from EK1, a pan-coronavirus fusion inhibitor, has displayed exceptional ability to inhibit RBD-ACE2 interaction in vivo [71]. Apart from this first pan-CoV fusion inhibitor (EK1C4), 136 new peptides have been developed from ACE2, of which the best peptide interacts with Arg 403, Tyr453, Gln 493, and Tyr505 in RBD, a striking similarity to A9 and A14 IRW analogs [28]. This serves as a lead drug design template for upcoming molecules as well. Likewise, a 23-aa peptide SBP1 (IEEQAKTFLDKFNHEAEDLFYQS) from ACE2 successfully stably binds to SARS-CoV-2 RBD in a manner similar to A9 and A14 [72]. Other peptides targeting this Spike RBD are being developed as well [73]. It is vital to note that the RBD region is also a critical target for neutralizing antibodies, another therapeutic used in COVID-19 treatment [74]. To further validate these findings, we conducted the pseudotyped lentiviral particles with the SARS-CoV-2 Spike assay as described in an earlier report (Figure 7A) [47]. These results confirmed the potential ability of A9 and A14 to lower SARS-CoV-2 viral load, particularly owing to the marked reduction in lentivirus particles. Our findings corroborate the other published data on bioactive compounds and SARS-CoV-2 lentivirus studies. For example, brazilin, TF-3, and curcumin can reduce the fusion of spike-expressing cells to the hACE2 in cells [75]. Other bioactive molecules with the ability against pseudo-virus particles include dimethoxycurcumin [76], peptides AYp28 [KKKKKKVEGFNCYFPLQS] and AYn1 [KKKKKKDKFNHEAEDLFY] [77], and tannic acid [78], to name a few. Similar to the studies on natural bioactives, we are unsure if A9 and A14 either destroy viral particles, or whether they via affecting membrane fluidity. One idea is that these peptides being lipophilic molecules, can provoke morphological changes in the cellular membrane, which may modify the lentivirus production in cells.

**Figure 9:**
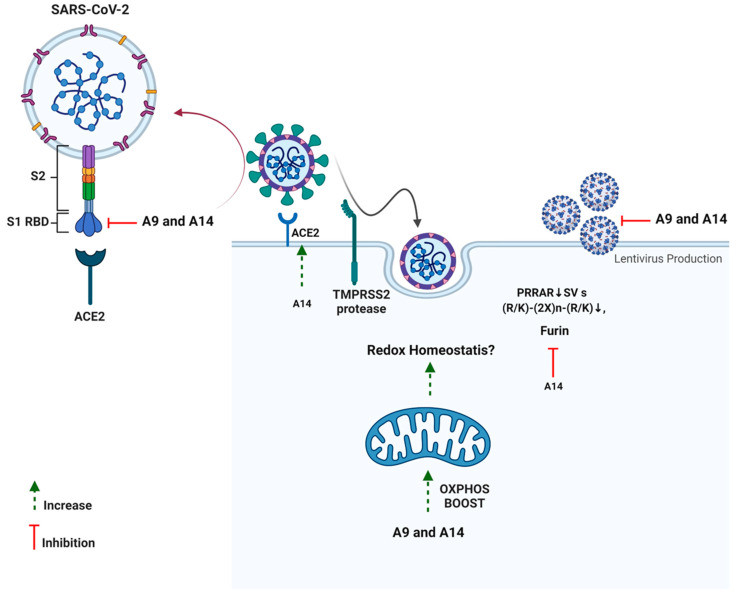
Schematic presentation of impact of peptides A9 and A14 against SARS-CoV-2.

Additionally, neither of the two analogs had any impact on TMPRSS2, a serine protease located on the host cell membrane, which promotes virus entry into the cell by activating the S protein [22]. This possibly indicates the fusion inhibitory activity of the analogs rather than any impact on spike activation. As discussed previously, SARS-CoV-2 S hides a furin cleavage site (682–685 residues) at the S1/S2 boundary, thus, providing another drug target [15]. Among the two analogs, analog A14 exhibited a strong ability to inhibit furin which can help in lowering the efficacy of SARS-CoV-2 transmission [79]. As furin is in the trans-Golgi network and activated by acidic pH, it is possible that A14 changes the pH of the cytoplasm or directly inhibits the enzyme [80]. In vitro studies indicate that cleavage occurs optimally at pH 6.0 via protonated His^69^, any change in pH can mitigate furin cleavage [80]. It would be interesting to see the impact of A14 on cellular pH in our upcoming studies. Interestingly, at a high concentration (100 µM), peptides A9 and A14 significantly inhibited the SARS-CoV-2 3CL Protease (aa1-306) in vitro (Figure S3). However, we ignored these results as the concentration of peptides was very high and the enzyme inhibition observed was <50%. Such results in enzyme analysis can be due to small molecule aggregates of candidate molecules in the assay plates. These aggregates may inhibit enzymes non-specifically at high micromolar concentrations in an aqueous solution via partial protein unfolding [81]. Overall, the two synthetic analogs of IRW managed to inhibit the SARS-CoV-2-ACE2 interaction, spike S1 overexpression, and furin along with the boost in OXPHOS levels in cells as well (Figure 9).

For future studies, we would like to study the impact of the two peptides A9 and A14 on viral replication using the live virus. The absence of this study is one of the critical drawbacks of our report. Next, we plan to have an animal trial to validate the findings from in vitro experiments. Given the current state of the pandemic, vaccines, both mRNA and adenovirus vector based, followed by immunotherapies, repurposed drugs, and nutraceutical adjuvants will be used against COVID-19. Repurposing of known modulators of SARS-CoV-2 targets can help quickly develop pharmaceutics against this global challenge. However, sincere efforts are required while conducting repurposing screens as many principles of drug development can be overlooked in the face of a global emergency. The use of cell lines such as HEK293T and VeroE6, often used in such screening can give false positives and negatives. Examples include chloroquine and camostat mesylate [82]. Secondly, minute changes and pharmacological effects observed need not be overinterpreted. Many of the in vitro screens fail as virus growth is logarithmical in nature. This applies to our findings as well as cellular attenuation of targets might not be replicated in live viral studies. Each analysis, for example, docking or in vitro results must complement each other to have any true antiviral impact. Likewise, RBD binding should be experimentally demonstrated using X-ray crystallography. Additionally, the results of spike protein expression need further analysis as viral proteins may be formed differently in a live virus system and use of different antibodies such as in our study and the original paper can give varying band weights [45]. Approved drug status or natural origin of drug source (including bioactive peptides) should not be falsely interpreted as a guarantee of non-toxic impact in the clinical settings of COVID-19. As COVID-19 is already lasting Aover years and will certainly be not the last global viral pandemic, extraordinary efforts including careful drug repurposing and design ought to be employed to meet challenges in view. 

## 5. Conclusions

Peptides as drug candidates hold multiple advantages including low toxicity, target specificity, and ease of synthesis. In this study, we designed structural analogs of a known ACE2 modulating tripeptide, IRW. Our experiments at in vitro, computational, and cellular levels identified its two analogs, A9 and A14, and potent inhibitors of SARS-CoV-2 RBD. These peptides mitigated the interaction of SARS-CoV-2 RBD-ACE2 and inhibited the overexpression of SARS-CoV-2 S1 protein in HEK293T cells. Conclusively, the reported peptides, following careful experimentation could serve as seeds for developing potent anti- SARS-CoV-2 drugs. 

## 6. Additional Disclosure

The authors of this publication do not advise or suggest any change in standard care and medications of COVID-19 patients. Our article solely reports two newly designed peptides for research purposes against SARS-CoV-2 infection. The content of this publication also does not necessarily reflect the views or policies of the affiliated institutions, nor does any mention of trade names, commercial products, nutraceuticals, or organizations imply endorsement by any pharmaceutical or food corporation or their products. Additionally, as per the BEI registration terms with our lab, all plasmids and cells obtained for conducting the SARS-CoV-2 Spike lentivirus study were autoclaved and disposed after completing the experiments required for this manuscript.

## Figures and Tables

**Table 1:** The binding energy of the peptides with ACE2 and the RBD.

Peptide	MM-GBSA Binding Energy	
	Spike RBD	ACE2
PM	−38.51	−30.47
A1	−45.29	−25.65
A2	−48.85	−29.47
A3	−48.75	−33.31
A4	−41.25	−26.47
A5	−47.39	−27.63
A6	−44.24	−23.88
A7	−47.39	−32.87
A8	−04.08	−23.51
A9	−57.22	−40.88
A10	−44.92	−20.47
A11	−48.80	−24.43
A12	−42.45	−23.34
A13	−40.22	−27.71
A14	−52.44	−35.51

## Data Availability

Not applicable.

## Supplementary Materials

The following supporting information can be downloaded at: https://www.mdpi.com/article/10.3390/pharmaceutics14102045/s1, Figure S1: Impact of constituent IRW di-peptides on SARS-CoV-2 S1 RBD-ACE2 interaction. An in vitro assay was conducted to assess the possible inhibitory impact of constituent IRW di-peptides (50 µM) on SARS-CoV-2 S1 RBD-ACE2 interaction. The results are expressed as percentage inhibition with respect vehicle control (nuclease free water). Data expressed as mean ± SEM of n = 3. *p* values were determined by Analysis by one-way ANOVA followed by Bonferroni’s post hoc test for the vehicle. ns: nonsignificant. Figure S2: Assessment of furin inhibitory potential of peptides A9 and A14. The analysis was conducted according to the methodology described. The results are expressed as the percentage furin inhibitory activity with respect vehicle control. Data expressed as mean ± SEM of n = 3. *p* values were determined by Analysis by one-way ANOVA followed by Bonferroni’s post hoc test for vehicle. **** indicates *p* < 0.001 versus vehicle. The error bar indicates S.D. of the mean value. µM: micromolar. Figure S3: Impact of peptides A9 and A14 on SARS-CoV-2 3CL Protease (aa1-306). The analysis was conducted according to the methodology described. The results are expressed as the percentage on SARS-CoV-2 3CL Protease (aa1-306) inhibitory activity with respect vehicle control. Data expressed as mean ± SEM of n = 6. *p* values were determined by Analysis by one-way ANOVA followed by Bonferroni’s post hoc test for vehicle. **** indicates *p* < 0.001 versus vehicle. The error bar indicates S.D. of the mean value. µM: micromolar. Figure S4: Histograms indicating florescence microscopy assessment of spike lentivirus inhibition potential of peptides A9 and A14. Histograms representing florescence intensity indicating lentivirus production in 293T-ACE2.TMPRSS2 cells transfected with the Spike lentiviral particles with the ZsGreen backbone in presence of A9 and A14 at 50 µM. The experiment was conducted n = 6 times independently. Figure S5: The interaction profile of A9 and A14 with ACE2. The amino acid residues 4 Å around the analogs are shown as lines. A9 and A14 are shown as sticks. Figure S6: The root mean square deviation curves of A9 and A14 relative to their initial configurations.
