# Supplementary material for: Structurally Modified Bioactive Peptide Inhibits SARS-CoV-2 Lentiviral Particles Expression
Source: Pharmaceutics. 2022 Sep 26;14(10):2045. doi: 10.3390/pharmaceutics14102045 (PMC9607082; doi:10.3390/pharmaceutics14102045)
Supplement: Supplementary file 1 [file pharmaceutics-14-02045-s001.zip › pharmaceutics-1903775-supplementary.pdf]

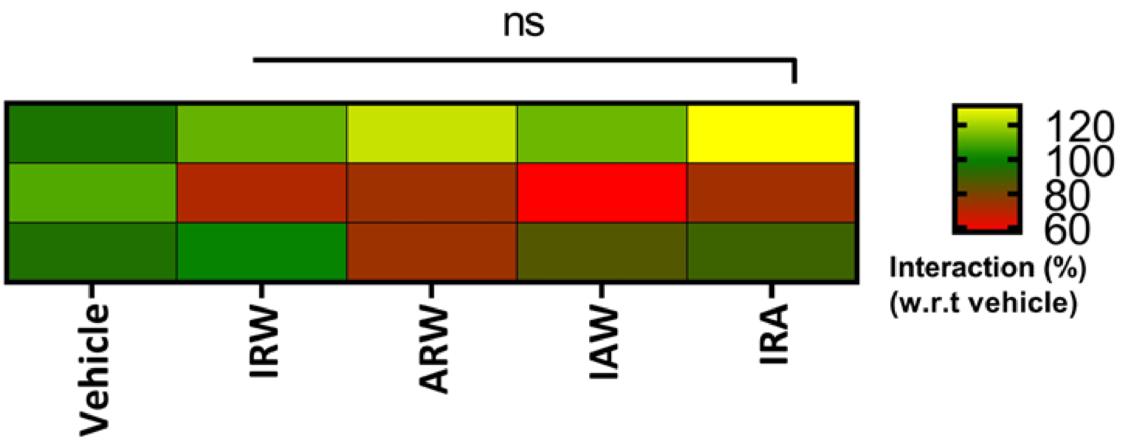

Figure S1. Impact of constituent IRW di-peptides on SARS-Cov-2 S1 RBD-ACE2 interaction. An in vitro assay was conducted to assess the possible inhibitory impact of constituent IRW di-peptides (50 μM) on SARS-Cov-2 S1 RBD-ACE2 interaction. The results are expressed as percentage inhibition with respect to the vehicle control (nuclease free water). Data expressed as mean ± SEM of n = 3. P values were determined by Analysis by one-way ANOVA followed by Bonferroni's post-hoc test for the vehicle. ns: nonsignificant.

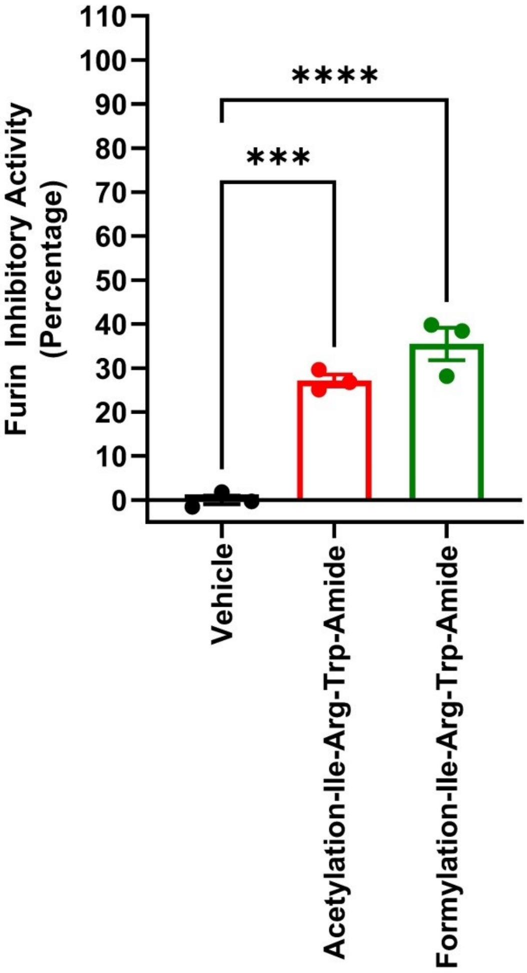

Figure S2. Assessment of furin inhibitory potential of peptides A9 (Acetylation-IRW-Amide) and A14 (Formylation IRW-Amide). The results are expressed as the percentage furin inhibitory activity with respect to the vehicle control. Data expressed as mean ± SEM of n=3. P values were determined by Analysis by one-way ANOVA followed by Bonferroni's post-hoc test for vehicle. *** indicates $p < 0.001$ and **** indicates $p < 0.0001$ versus vehicle. The error bar indicates S.D. of the mean value. The assay was conducted at the peptide concentration of 50 µM. µM: micromolar.

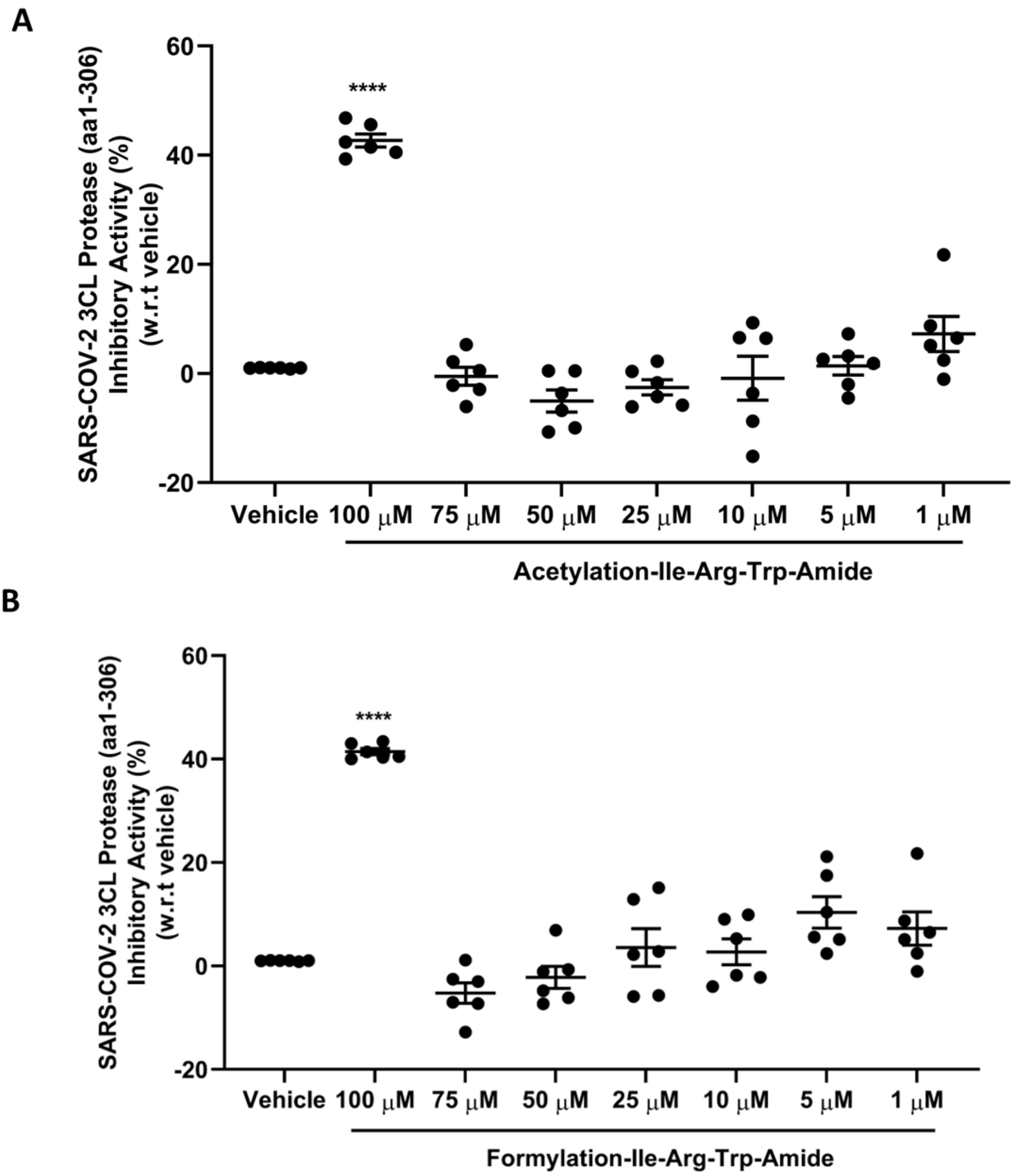

Figure S3. Impact of the selected peptides (**A**) Acetylation-IRW-Amide and (**B**) Formylation IRW-Amide on SARS-COV-2 3CL Protease (aa1-306) activity at various concentrations. The results are expressed as the percentage on SARS-COV-2 3CL Protease (aa1-306) inhibitory activity with respect to the vehicle control. Data expressed as mean ± SEM of n = 6. P values were determined by Analysis by one-way ANOVA followed by Bonferroni's post-hoc test for vehicle. **** indicates $p < 0.0001$ versus vehicle. The error bar indicates S.D. of the mean value. μM: micromolar.

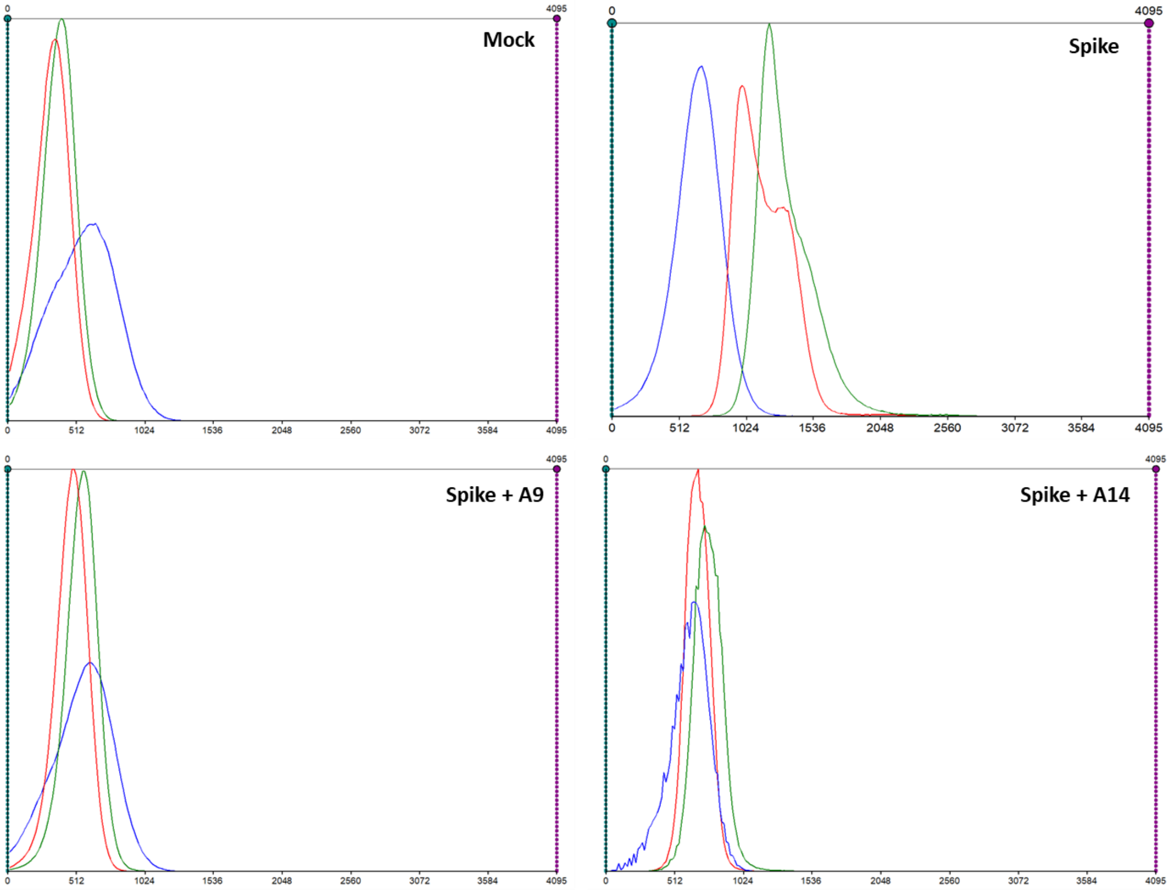

Figure S4. Histograms indicating florescence microscopy assessment of spike lentivirus inhibition potential of peptides A9 and A14. Histograms representing florescence intensity indicating lentivirus production in 293T-ACE2.TMPRSS2 cells transfected with the Spike lentiviral particles with the ZsGreen backbone in presence of A9 and A14 at 50 μM. The experiment was conducted n = 6 times independently.

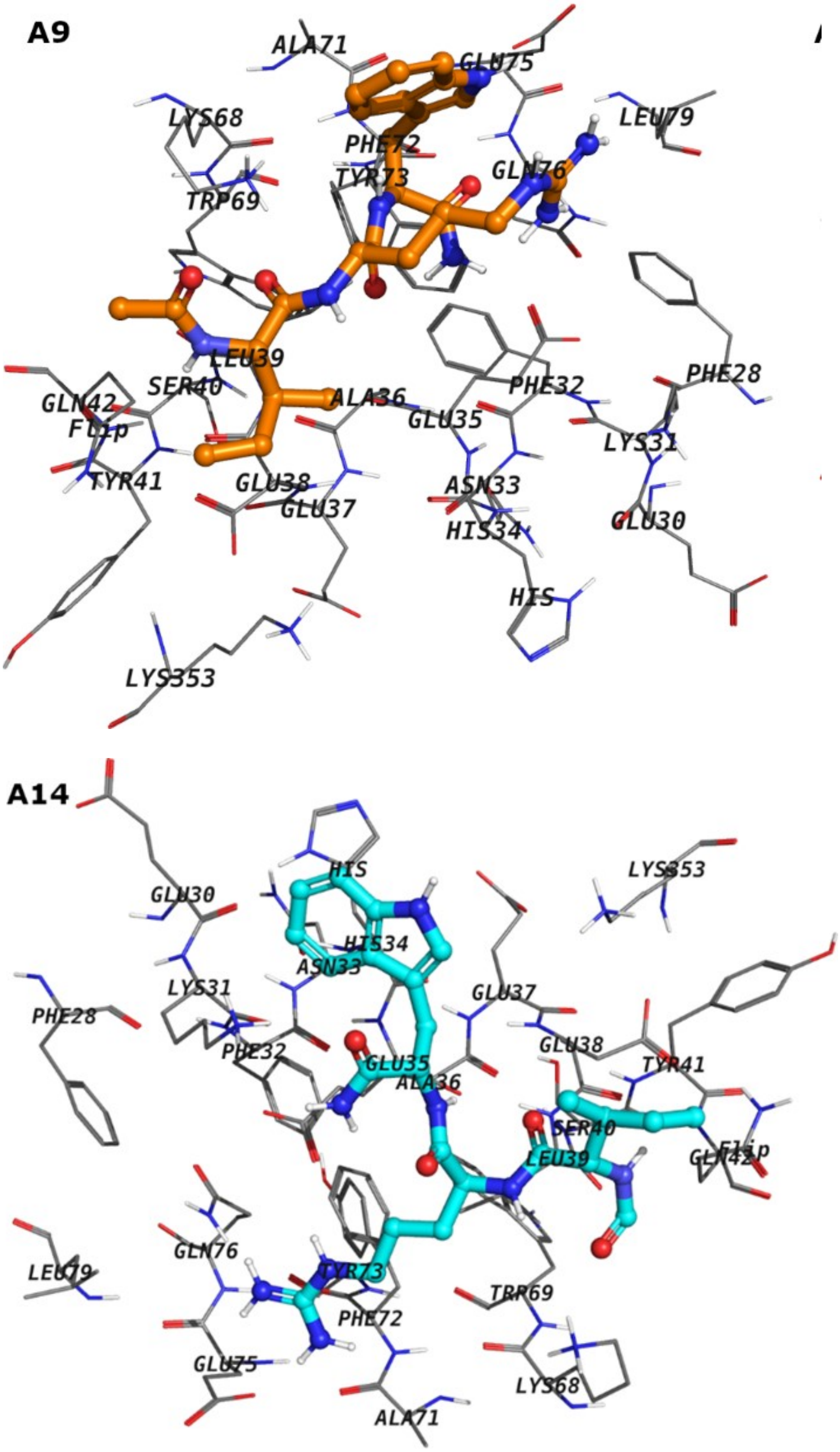

Figure S5. The interaction profile of A9 and A14 with ACE2. The amino acid residues 4 Å around the analogs are shown as lines. A9 and A14 are shown as sticks.

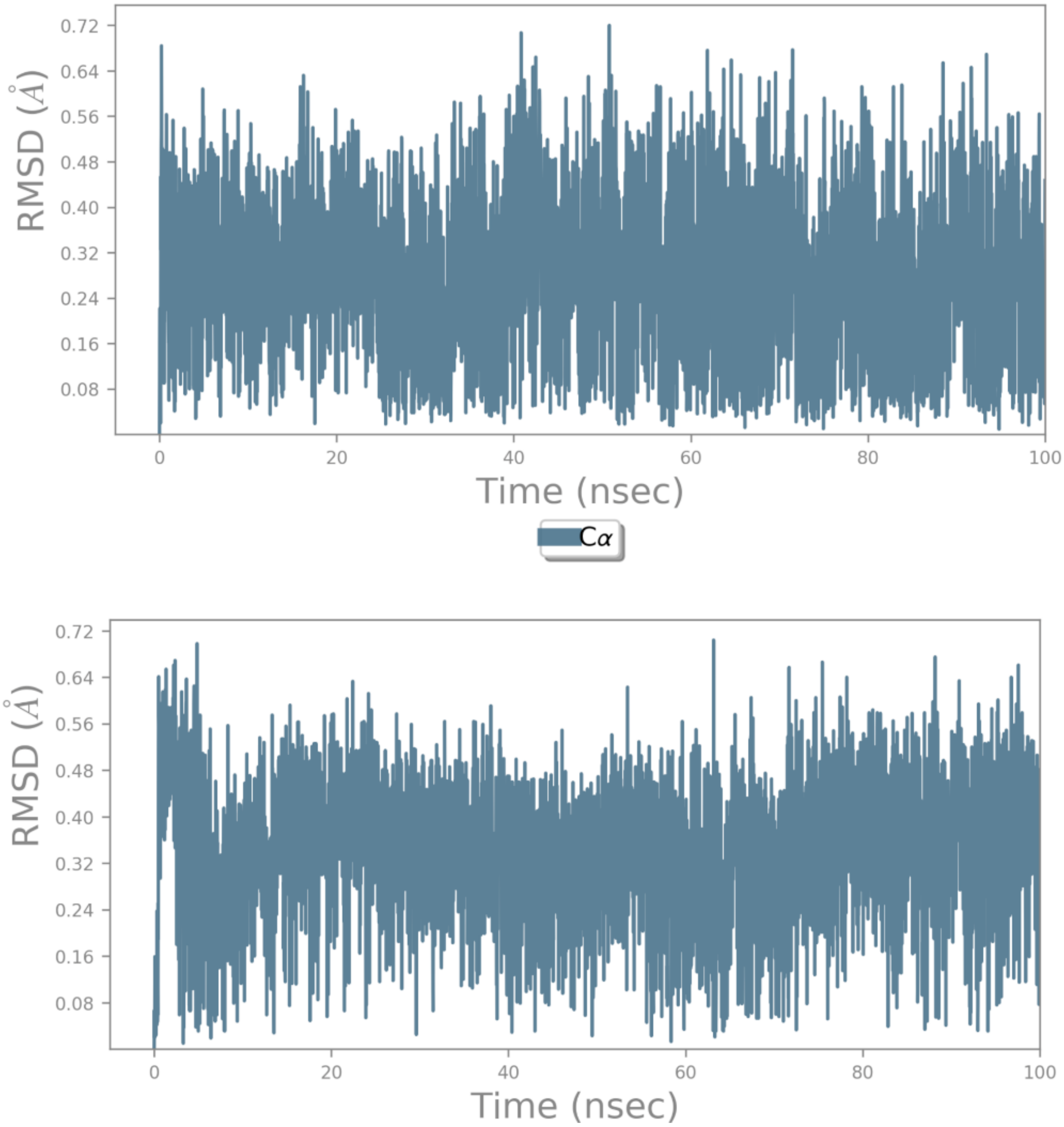

Figure S6. The root mean square deviation curves of A9 (Acetylation-IRW-Amide) and A14 (Formylation IRW-Amide) relative to their initial configurations.
